# Different biomarker ratios in peripheral blood have limited value in diagnosing periprosthetic joint infection after total joint arthroplasty: a single-center, retrospective study

**DOI:** 10.1186/s12891-024-07499-7

**Published:** 2024-05-13

**Authors:** Lei Deng, Jie Wang, Guang-ya Yang, Ying-zhou Hou, Ke-wei Li, Bo Sun, Shao-hua Wang

**Affiliations:** 1 The First School of Clinical Medicine, Henan University of Chinese Medicine, Zhengzhou, 450052 People’s Republic of China; 2 Department of Orthopaedic, Beijing ChaoYang Hospital, Capital Medical University, Beijing, 100020 People’s Republic of China; 3 Department of Joint Surgery, Zhengzhou Orthopaedic Hospital, Zhengzhou, 450052 People’s Republic of China

**Keywords:** Artificial joint replacement, Periprosthetic joint infection, Biomarkers, Peripheral blood, Diagnosis

## Abstract

**Background:**

Periprosthetic joint infection (PJI) is a severe complication that can occur after total joint arthroplasty (TJA). The timely and accurate diagnosis of PJI is the key to treatment. This study investigated the diagnostic value of platelet to lymphocyte ratio (PLR), platelet count to mean platelet volume ratio (PVR), neutrophil to lymphocyte ratio (NLR) and monocyte to lymphocyte ratio (MLR) in PJI after total knee arthroplasty (TKA) and total hip arthroplasty (THA).

**Methods:**

We performed a retrospective analysis of the patients who underwent revision hip or knee arthroplasty at our Institute between June 2015 and June 2020. Of the 187 patients reviewed, 168 were included in the study. According to the diagnostic criteria of the Musculoskeletal Infection Society (MSIS), 58 patients were in the PJI group, and 110 patients were in the aseptic loosening (AL) group. We recorded and compared the preoperative peripheral blood white blood cell (WBC) count, platelet count (PLT), erythrocyte sedimentation rate (ESR), C-reactive protein (CRP), PLR, PVR, NLR, and MLR in both groups. The diagnostic performance of the WBC, PLT, PLR, PVR, NLR, and MLR individually and in combination with the ESR and CRP for PJI diagnosis was evaluated by receiver operating characteristic (ROC) curves, and the sensitivity, specificity, positive predictive value, and negative predictive value were calculated.

**Results:**

Compared to those in the AL group, the mean WBC, PLT, ESR, CRP, PLR, PVR, NLR, and MLR in the peripheral blood of the PJI group were significantly greater (*P* < 0.05). The analysis of the ROC curve revealed that the ESR, CRP, PLR, PVR, NLR, and MLR in peripheral blood had moderate effectiveness in diagnosing PJI, with area under the curve (AUC) values of 0.760 (95% CI: 0.688–0.823), 0.758 (95% CI: 0.687–0.821), 0.714 (95% CI: 0.639–0.781), 0.709 (95% CI: 0.634–0.777), 0.723 (95% CI: 0.649–0.789), and 0.728 (95% CI: 0.654–0.793), respectively. Conversely, the WBC and PLT counts demonstrated poor diagnostic value for PJI, with AUC values of 0.578 (95% CI: 0.499–0.653) and 0.694 (95% CI: 0.619–0.763), respectively. The results of the prediction model calculations revealed that the combined AUC of the WBC, PLT, ESR, CRP, PLR, PVR, NLR, and MLR was the highest at 0.853 (95% CI, 0.790–0.909), indicating good value in the diagnosis of PJI, with a sensitivity of 82.8% and a specificity of 72.7%. Moreover, the novel composite of parameters improved the accuracy and reliability in diagnosing PJI compared to the traditional biomarkers ESR and CRP (*P* = 0.015).

**Conclusion:**

Our study suggested that the diagnostic value of the peripheral blood biomarkers PLR, PVR, NLR, and MLR for diagnosing PJI is limited and not superior to that of the ESR or CRP. However, when the WBC, PLT, ESR, CRP, PLR, PVR, NLR, and MLR are combined, the diagnostic performance of PJI in TJA patients can be improved.

## Introduction

Currently, the diagnosis of periprosthetic joint infection (PJI) still relies heavily on the culture of pathogenic microorganisms, which is widely considered the "gold standard" [1]. However, with the emergence of specific pathogen infections such as mycobacteria, Brucella, and fungi and the formation of bacterial biofilms, these factors may diminish the detection rate of pathogens and exacerbate the challenges in PJI diagnosis and treatment [2–4]. Approximately 5%-42% of PJI patients are estimated to demonstrate negative results for pathogenic microorganisms [5]. To increase the accuracy of diagnosing PJI, clinicians must integrate clinical manifestations, imaging evidence, and a variety of laboratory tests [1–3, 6, 7]. Classic biomarkers of human inflammation, C-reactive protein (CRP) and the erythrocyte sedimentation rate (ESR), have been extensively employed to assist in PJI diagnosis [8]. Sigmund et al. demonstrated that the serum ESR has a sensitivity range of 33% to 95% and a specificity range of 60% to 100%, while the sensitivity range of CRP is from 62 to 100%, and the specificity range is from 64 to 96% [9]. Although the ESR and CRP can be useful in assisting with the diagnosis of PJI, their diagnostic accuracy may not always meet the needs of clinical physicians. In recent years, several researchers have proposed innovative molecular biological methods, such as polymerase chain reaction (PCR), multiplex PCR, mass spectrometric analysis, and next-generation sequencing (NGS), as new approaches for diagnosing PJI [10, 11]. Although these methods can further improve the accuracy of PJI diagnosis, they present significant challenges for implementation in primary hospitals due to their professional operation and high cost [10–12].

As a conventional diagnostic biomarker for hospitalized patients, the peripheral blood test is a simple, cost-effective, and widely adaptable test for various infectious diseases. Several biomarkers derived from peripheral blood, such as the ESR, CRP, white blood cell (WBC) count, and leukocyte classification, can assist medical professionals in determining inflammatory status, severity of infection, and type of infection within the body [9, 13]. Recent studies have revealed that changes in the PLR, PVR, NLR and MLR are closely related to inflammatory or infectious status in the body [13, 14]. It is reported that the diagnostic accuracy of PVR in patients with fracture-related infection was no less significant than that of ESR or CRP, suggesting that PVR could function as an auxiliary diagnostic biomarker of fracture-caused infection [15]. The PLR and NLR have been found to have good diagnostic value for predicting surgical site infection, while the MLR and NLR demonstrate significant variation in feverish patients due to bacterial infections [13–15]. A retrospective study also revealed that the PLR and NLR in the PJI group were significantly greater than those in the aseptic loosening (AL) group [16]. These different biomarker ratios reflect the severity of the infection and make an early diagnosis of the disease. Therefore, further study of the correlation between these biomarkers and PJI is significant.

To explore the diagnostic value of inexpensive peripheral blood biomarkers in periprosthetic infections, we conducted a further analysis of these biomarkers in this single-center retrospective study. We compared these different biomarker ratios with the classical serum biomarkers ESR and CRP to explore the importance of PVR, the PLR, the NLR and the MLR in the diagnosis of PJI after TJA. Furthermore, we combined these biomarkers with the ESR and CRP to evaluate the potential value of each combination in diagnosing PJI.

## Materials and methods

### Study design

This retrospective study analyzed the data of 187 patients who required revision surgery due to unexplained fever and pain after total knee arthroplasty (TKA) and total hip arthroplasty (THA) from June 2015 to June 2020 in the Department of Joint Surgery of Zhengzhou Orthopedic Hospital. The exclusion criteria for patients were as follows: (i) Patients with immune system diseases such as rheumatoid arthritis (RA) or ankylosing spondylitis (AS); (ii) Patients with combined systemic infectious diseases or infections at other sites; (iii) Patients received revision surgery for periprosthetic fractures or prosthetic dislocations; (iv) Severe liver disease or malignant tumours; (v) Lacked complete clinical data or refused to participate in this study. After applying the exclusion criteria, a final cohort of 168 participants was included in the analysis. PJI was diagnosed according to the criteria established by the Musculoskeletal Infection Society (MSIS) [17], including major and minor criteria: (1) Major criteria: 2 positive periprosthetic cultures with phenotypically identical organisms or a sinus tract communicating with the joint; (2) Minor criteria: (i) Elevated serum CRP and ESR; (ii) Elevated synovial fluid WBC count or leukocyte esterase test +  + ; (iii) Elevated synovial fluid polymorphonuclear neutrophil percentage (PMN%); (iv) Positive histological analysis of periprosthetic tissue; (v) A single positive culture. The diagnosis of PJI requires that one of the major or three of the five minor criteria be met. The Board Review Committee of Zhengzhou Orthopaedic Hospital approved our study.

### Data collection

On the day of admission, nurses collected fasting cubital vein blood specimens 10 ml from all patients and sent them to our hospital’s laboratory for analysis within 1 h. Peripheral blood routine examination is performed utilizing the Sysmex XN-9000 Hematology Analyzer (Japan), ESR testing is performed utilizing special ESR tubes compatible with the VITAL Monitor-100 system (Italy), and CRP testing is conducted using the HITACHI-7600 automated chemistry analyzer (Japan). Patient general information, including age, sex, body mass index (BMI), preoperative peripheral blood white blood cell (WBC) count, platelet count (PLT), ESR, CRP, PLR, PVR, NLR and MLR, was recorded for both groups based on our hospital's electronic medical record system. In addition, in this study, we collected joint fluid and periprosthetic tissue samples (at least three locations) from patients with diagnosed or suspected PJI. We classified and counted the WBC in the joint fluid, performed aerobic and anaerobic cultures on the samples, and conducted pathological analysis on the periprosthetic tissue.

### Statistical methods

Statistical analysis and graphing were conducted using SPSS 24.0 (IBM, Armonk, NY, USA), MedCalc 19.0.4 (MedCalc Software by Ostend, Belgium), and GraphPad Prism 8.0.2. Continuous variables are expressed as the mean ± standard deviation (SD), and categorical variables are expressed as frequencies or percentages. The Mann–Whitney U test was used to compare continuous variables between two groups, and the chi-square test was adopted to analyze categorical variables. The values of the PLR, PVR, NLR, and MLR, individually or in combination with other biomarkers, in assisting in diagnosing PJI were evaluated by constructing receiver operating characteristic (ROC) curves. The optimum cut-off value for all tested biomarkers was determined based on the Youden index, and the area under the curve (AUC), sensitivity, specificity, positive predictive value (PPV), negative predictive value (NPV), positive likelihood ratio (+ LR) and negative likelihood ratio (-LR) were calculated for the individual or combined use of each indicator. The AUC values ranging from 0.900 to 1.000 were defined as excellent, 0.800 to 0.899 as good, 0.700 to 0.799 as medium, 0.600 to 0.699 as poor, and 0.500 to 0.599 as having no diagnostic ability [18]. The Delong test was used to compare the differences in the AUC. A *P* value < 0.05 was considered statistically significant, multiple comparisons were conducted using the Bonferroni method to adjust the level of α.

## Results

### Basic characteristics of all patents in the PJI group and AL group

**Fig. 1:**
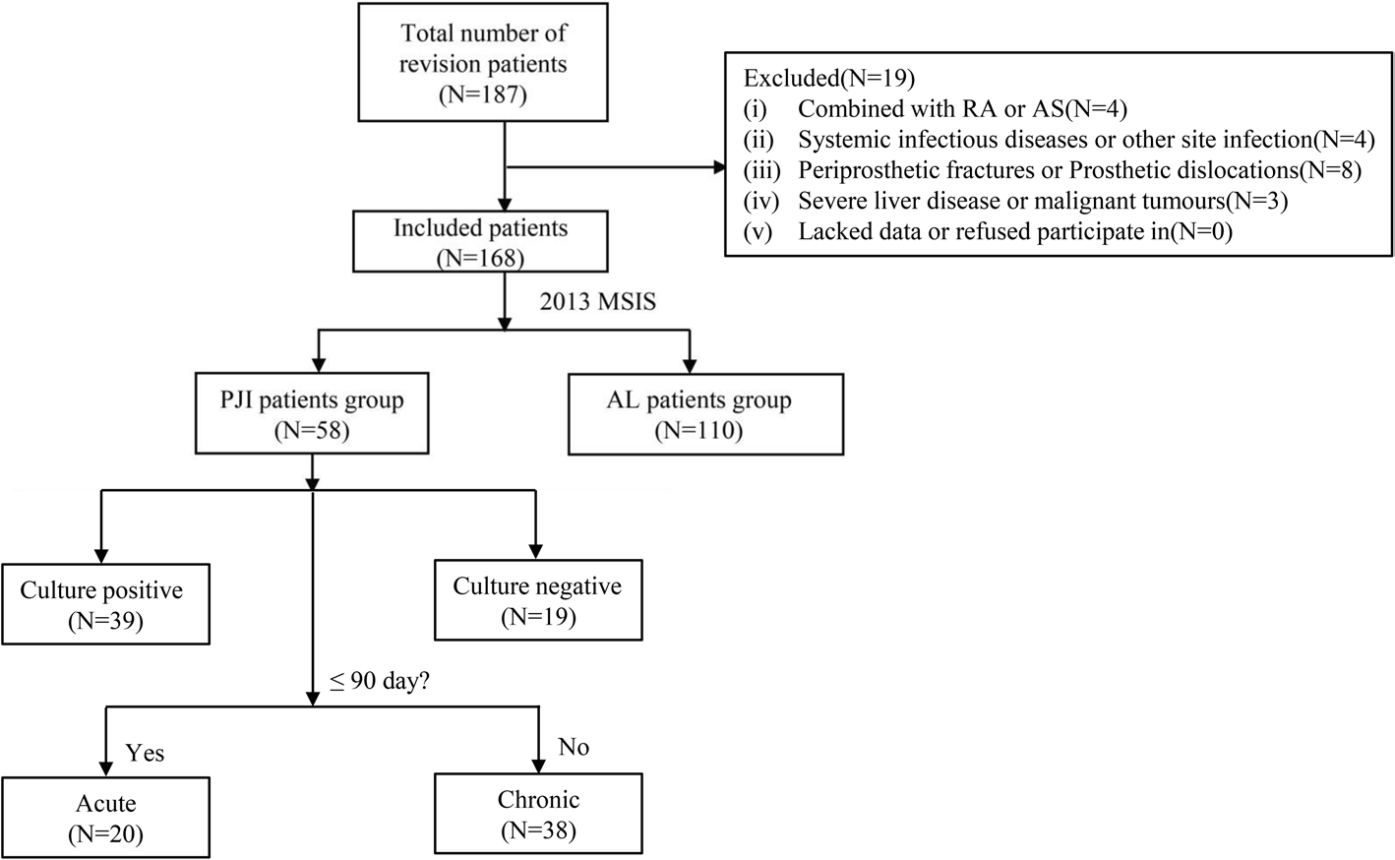
Patient inclusion and exclusion procedures

The patients were stratified into two groups based on the diagnostic criteria established by the MSIS [17], with 58 patients allocated to the PJI group and 110 patients assigned to the AL group. According to the result of pathogenic microorganism culture and the time of their last surgery, patients were divided into different PJI subgroups (Fig. 1).

The following table (Table 1) compares basic characteristics between the PJI and AL groups. No statistically significant differences were observed in critical demographic information, including age, sex, BMI, surgical side, and so on (*P* > 0.05).

**Table 1:** Basic characteristics of all patents in the PJI group and AL group

	ALL (*N* = 168)	PJI Group (*N* = 58)	AL Group (*N* = 110)	*P* value
Age (years) (mean ± SD)	67.14 ± 8.23	68.64 ± 8.05	66.35 ± 8.25	0.086
Gender				0.138
Male (%)	71 (42.3%)	20 (34.5%)	51 (46.4%)	
Female (%)	97 (57.7%)	38 (65.5%)	59 (53.6%)	
BMI (kg/m^2^) (mean ± SD)	25.49 ± 1.65	25.80 ± 1.51	25.40 ± 1.59	0.113
Site				
Left (%)	82 (48.8%)	33 (56.9%)	49 (44.6%)	0.128
Right (%)	86 (51.2%)	25 (43.1%)	61 (55.4%)	
Joint				
Knee (%)	103 (61.3%)	37 (63.8%)	66 (60.0%)	0.631
Hip (%)	65 (38.7%)	21 (36.2%)	44 (40.0%)	

### Comparison of peripheral blood biomarkers results between the two groups

**Fig. 2:**
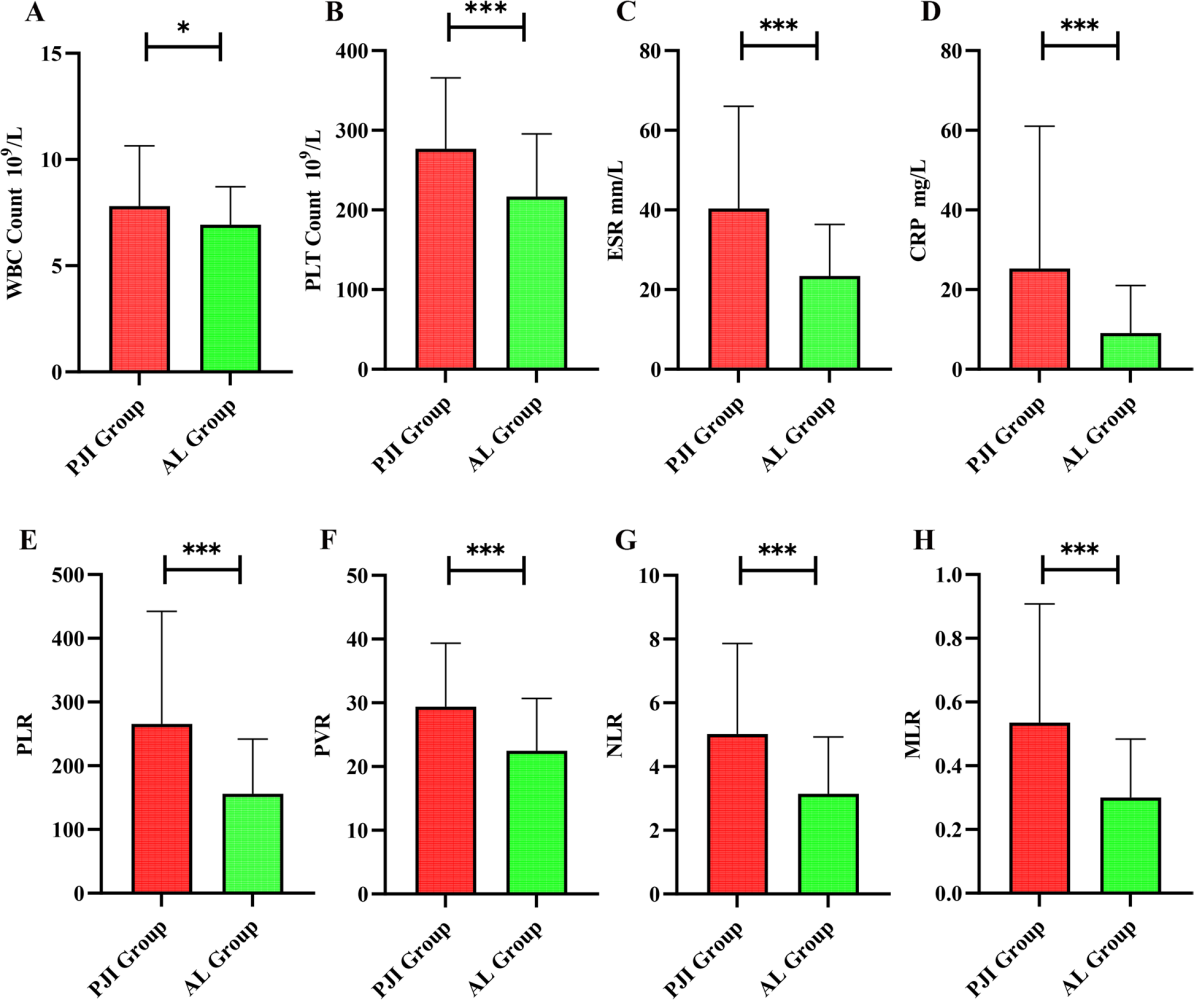
Comparison of peripheral blood biomarker results between the PJI group and the AL group. Figure Note: **A** white blood cell count (WBC); **B** platelet count (PLT); **C** Erythrocyte sedimentation rate (ESR); **D** C-reactive protein (CRP); **E** Platelet to lymphocyte ratio (PLR); **F** Platelet count to mean platelet volume ratio (PVR); **G** Neutrophil to lymphocyte ratio (NLR); **H** Monocyte to lymphocyte ratio (MLR). ***P < 0.001

The preoperative peripheral blood WBC count, PLT, ESR, CRP, PLR, PVR, NLR, and MLR were compared between the PJI and AL groups. The Table 2 and Fig. 2 show that the patients in the PJI group had significantly higher levels of WBC count (7.81 ± 2.83 vs 6.93 ± 1.79) than the AL group (*P* < 0.05), and also the PLT (276.67 ± 89.10 vs 216.89 ± 78.45), ESR (40.36 ± 25.65 vs 23.41 ± 12.96), CRP (25.29 ± 35.71 vs 9.10 ± 11.96), PLR (265.55 ± 176.79 vs 156.14 ± 85.83), PVR (29.41 ± 9.94 vs 22.49 ± 8.19), NLR (5.02 ± 2.84 vs 3.15 ± 1.78), and MLR (0.54 ± 0.37 vs 0.30 ± 0.18) than AL group (*P* < 0.001) (Fig. 2, Table 2).

**Table 2:** Results of peripheral blood biomarkers in PJI group and AL group

Laboratory examination	PJI Group (*N* = 58)		AL Group (*N* = 110)		*P* value
	(mean ± SD)	*M* (*P*_25_, *P*_75_)	(mean ± SD)	*M* (*P*_25_, *P*_75_)	
WBC (*109/L) *M* (*P*_25_, *P*_75_)	7.81 ± 2.83	7.1 (5.95, 8.86)	6.93 ± 1.79	6.76 (5.59, 7.80)	0.015
PLT (*109/L) (mean ± SD)	276.67 ± 89.10	266.0 (217.5, 343.0)	216.89 ± 78.45	221.5 (156.0,265.0)	< 0.001
ESR (mm/h)	40.36 ± 25.65	33.5 (23.75, 46.25)	23.41 ± 12.96	21 (15,29)	< 0.001
CRP (mg/L)	25.29 ± 35.71	16.31 (7.67, 26.97)	9.10 ± 11.96	4.54 (1.37, 11.88)	< 0.001
PLR	265.55 ± 176.79	222.17 (138.69, 348.04)	156.14 ± 85.83	133.32 (97.78, 207.21)	< 0.001
PVR	29.41 ± 9.94	29.05 (22.07, 36.88)	22.49 ± 8.19	22.725 (16.02, 27.18)	< 0.001
NLR	5.02 ± 2.84	4.32 (2.63, 6.07)	3.15 ± 1.78	2.62 (1.74, 3.97)	< 0.001
MLR	0.54 ± 0.37	0.5 (0.24, 0.63)	0.30 ± 0.18	0.24 (0.17, 0.39)	< 0.001

### ROC curves analysis for individual peripheral blood biomarkers in PJI diagnosis

**Fig. 3:**
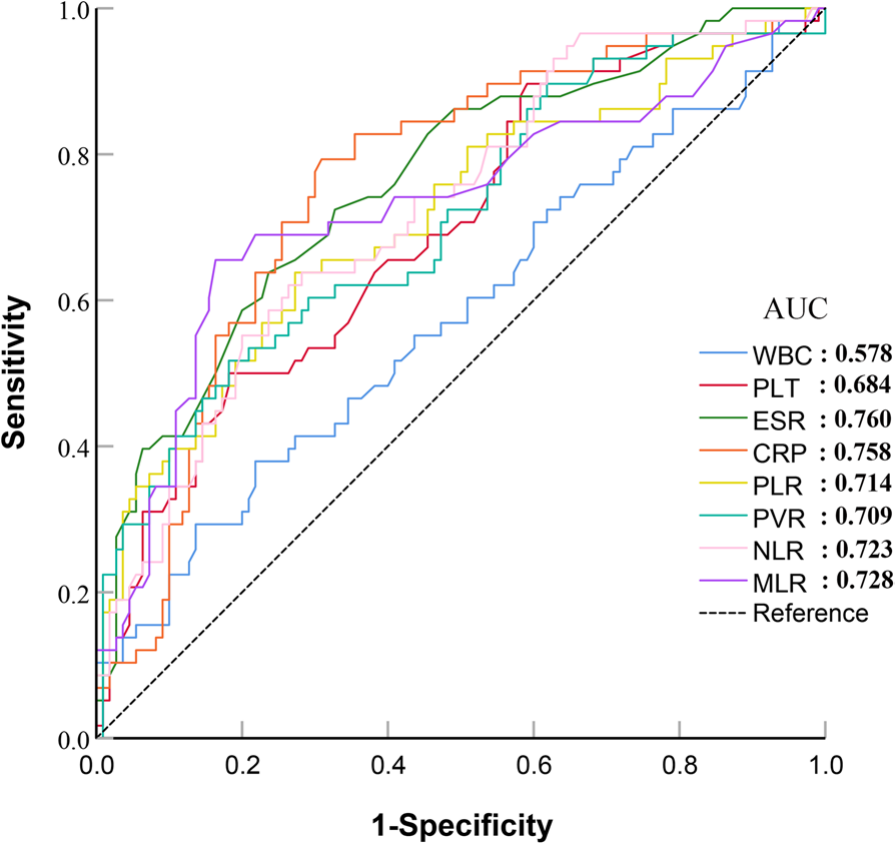
Results of ROC analysis of individual peripheral blood biomarkers

The diagnostic value of individual peripheral blood biomarkers was analyzed by constructing ROC curves. The AUC ranked from highest to lowest were ESR (AUC = 0.760) > CRP (AUC = 0.758) > MLR (AUC = 0.728) > NLR (AUC = 0.723) > PLR (AUC = 0.714) > PVR (AUC = 0.709) > PLT (AUC = 0.694) > WBC (AUC = 0.578). Our study results indicate that WBC and PLT have poor diagnostic values for PJI; ESR, CRP, PLR, PVR, NLR, and MLR have medium diagnostic values for PJI; and the PLR, PVR, NLR, and MLR are not superior to the ESR or CRP in diagnosing PJI (Fig. 3).

After evaluating the sensitivity and specificity of the indicators with an AUC greater than 0.700, it was determined that the optimal cut-off for the ESR was 29 mm/h, with a sensitivity of 63.8% and specificity of 76.4%. The optimal cut-off for the CRP was 7.53 mg/L, with a sensitivity of 79.3% and a specificity of 69.1%. The optimal cut-off for the PLR was 178.92, with a sensitivity of 63.8% and a specificity of 72.7%. The optimal cut-off for PVR was 28.27, with a sensitivity of 51.7% and a specificity of 81.8%. The optimal cut-off for the NLR was 3.79, with a sensitivity of 62.1% and a specificity of 73.6%. Finally, the optimal cut-off for the MLR was 0.43, with a sensitivity of 65.5% and a specificity of 83.6% (Fig. 3, Table 3).

**Table 3:** Diagnostic value of individual peripheral blood biomarkers in PJI

Laboratory examination	AUC (95% CI)	Youden Index	Cut off	P* value	Sensitivity	Specificity	PPV	NPV	+ LR	-LR
WBC	0.578 (0.499–0.653)	0.161	8.03	0.004	37.9%	78.2%	47.8%	70.5%	173.9%	79.4%
PLT	0.694 (0.619–0.763)	0.318	273	0.230	50.0%	81.8%	59.2%	75.6%	274.7%	61.1%
ESR	0.760 (0.688–0.823)	0.402	29	-	63.8%	76.4%	58.7%	80.0%	270.3%	47.4%
CRP	0.758 (0.687–0.821)	0.484	7.53	0.971	79.3%	69.1%	57.5%	86.4%	256.6%	30.0%
PLR	0.714 (0.639–0.781)	0.365	178.92	0.334	63.8%	72.7%	55.2%	79.2%	233.7%	49.8%
PVR	0.709 (0.634–0.777)	0.335	28.27	0.350	51.7%	81.8%	60.0%	76.3%	284.1%	59.0%
NLR	0.723 (0.649–0.789)	0.357	3.79	0.426	62.1%	73.6%	55.4%	78.6%	235.2%	51.5%
MLR	0.728 (0.654–0.793)	0.492	0.43	0.466	65.5%	83.6%	67.9%	82.1%	399.4%	41.3%

*PPV* positive predictive value, *NPV* negative predictive value, + *LR* positive likelihood ratio, *-LR* negative likelihood ratio

^*^Comparison with the AUC of ESR

### ROC curves analysis for combined peripheral blood biomarkers in PJI diagnosis

**Fig. 4:**
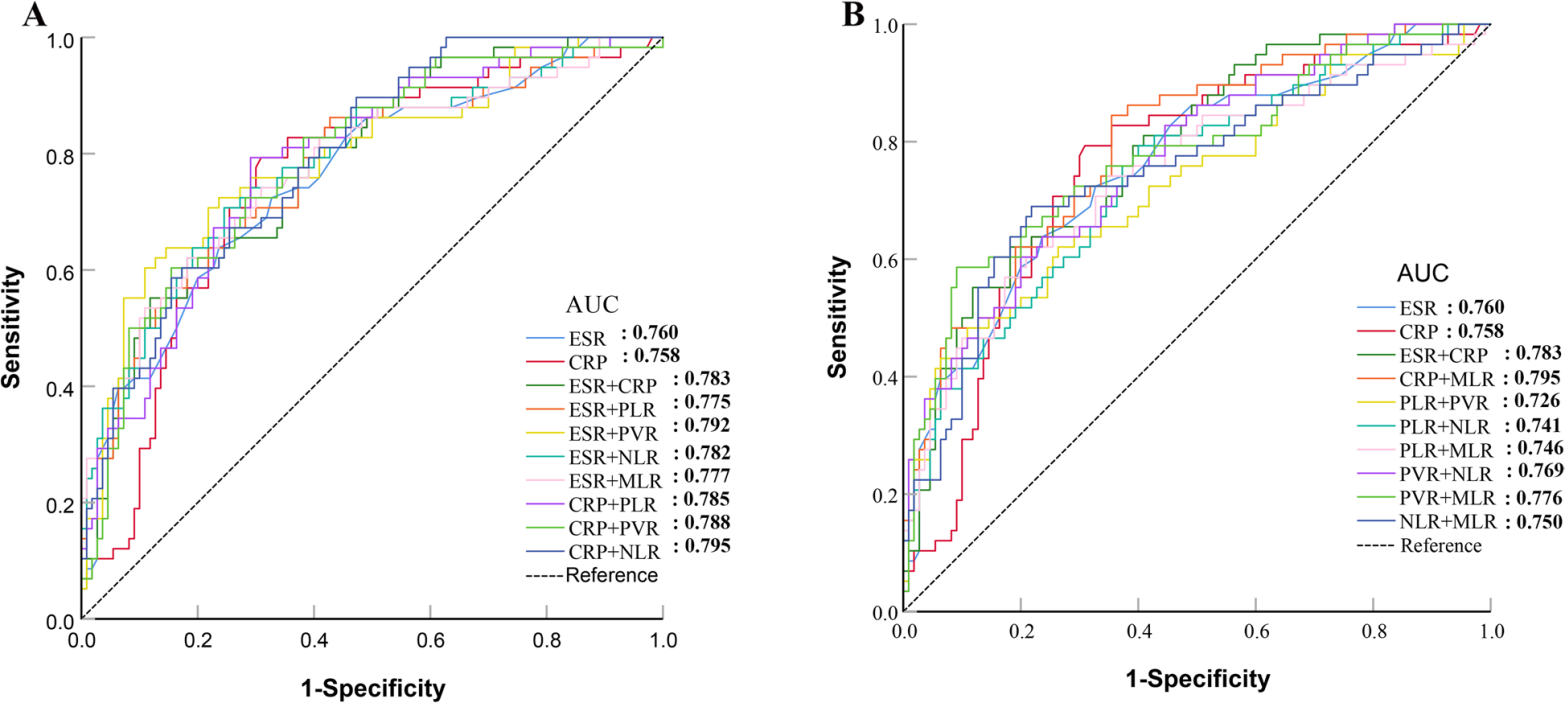
Results of the ROC analysis of patients combination with two peripheral blood biomarkers

**Fig. 5:**
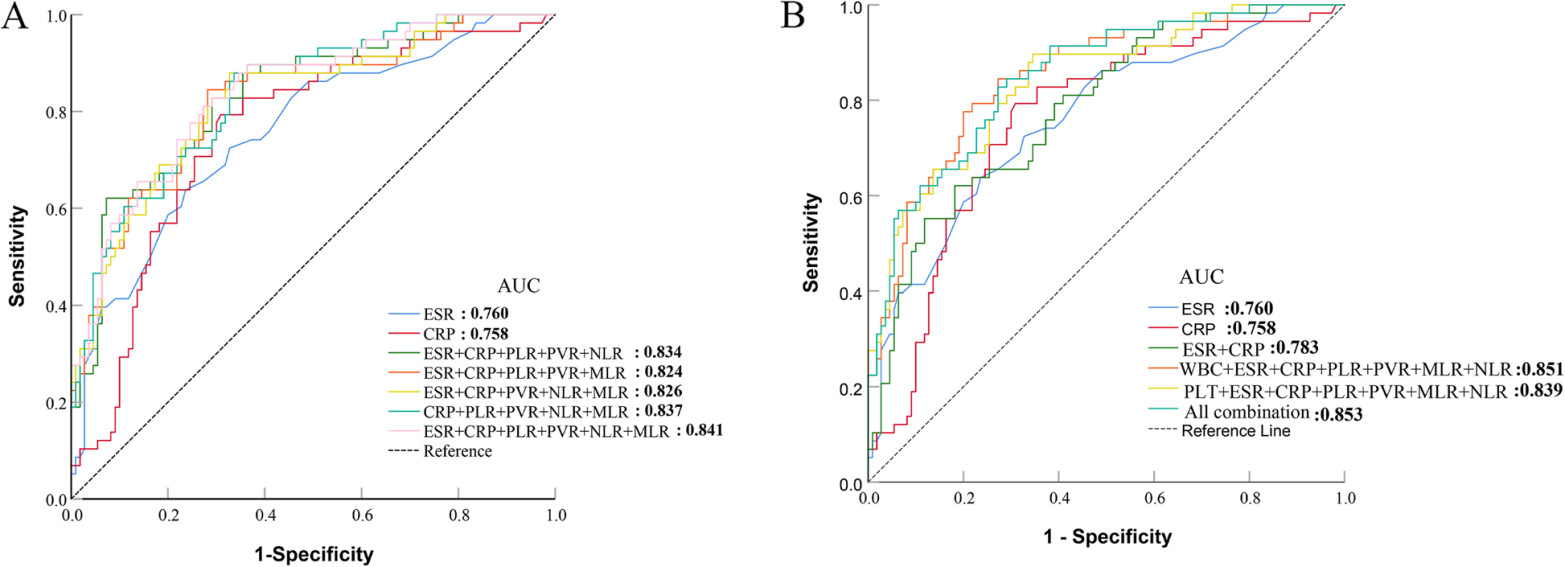
Results of the ROC analysis of patients in combination with five or all peripheral blood biomarkers

In comparing a range of combinations involving the ESR, CRP, PLR, PVR, NLR, and MLR in peripheral blood with ESR and CRP separately, the ROC curve results indicate that thoroughly combining all the biomarkers exhibits the highest AUC (0.853 (95% CI, 0.790–0.909). The novel combination demonstrates favorable sensitivity and specificity, 82.8% and 72.7%, respectively. In contrast with individual and combined assessments of CRP and ESR, this new combined approach showed substantial diagnostic value (*P* = 0.015) (Figs. 4 and 5, Tables 4 and 5).

**Table 4:** Diagnostic value of the combination of two peripheral blood biomarkers for PJI

Different combination	AUC (95% CI)	*P* value*	*P* Value^&^	*P* value^#^	Sensitivity	Specificity	PPV	NPV	+ LR	-LR
ESR	0.760 (0.688–0.823)	-	0.971	0.152	63.8%	76.4%	58.7%	80.0%	270.3%	47.4%
CRP	0.758 (0.687–0.821)	0.971	-	0.483	79.3%	69.1%	57.5%	86.4%	256.6%	30.0%
**The combination of two biomarkers**										
ESR + CRP	0.783 (0.713–0.843)	0.152	0.483	-	62.1%	81.8%	64.3%	80.4%	341.4%	46.4%
ESR + PLR	0.775 (0.704–0.836)	0.551	0.738	0.775	86.2%	57.3%	51.5%	88.7%	201.8%	24.1%
ESR + PVR	0.792 (0.723–0.851)	0.286	0.485	0.784	60.3%	89.1%	74.5%	81.0%	553.1%	44.5%
ESR + NLR	0.782 (0.712–0.842)	0.317	0.643	0.979	70.7%	75.5%	60.3%	83.0%	287.9%	38.8%
ESR + MLR	0.777 (0.707–0.838)	0.467	0.712	0.833	62.1%	81.8%	64.3%	80.4%	341.4%	46.4%
CRP + PLR	0.785 (0.715–0.845)	0.539	0.428	0.954	79.3%	70.9%	59.0%	86.7%	272.6%	29.2%
CRP + PVR	0.788 (0.718–0.847)	0.539	0.372	0.897	60.3%	84.6%	67.3%	80.2%	390.6%	46.9%
CRP + NLR	0.795 (0.726–0.853)	0.352	0.320	0.696	58.6%	84.6%	66.7%	79.5%	379.4%	48.9%
CRP + MLR	0.795 (0.726–0.853)	0.340	0.347	0.692	84.5%	64.6%	55.7%	88.7%	238.3%	24.0%
PLR + PVR	0.726 (0.652–0.792)	0.501	0.567	0.259	48.3%	89.1%	70.0%	76.6%	442.5%	58.1%
PLR + NLR	0.741 (0.668–0.806)	0.680	0.762	0.358	79.3%	60.0%	51.1%	84.6%	198.3%	34.5%
PLR + MLR	0.746 (0.673–0.810)	0.738	0.823	0.396	56.9%	82.7%	63.5%	78.5%	329.5%	52.1%
PVR + NLR	0.769 (0.698–0.830)	0.856	0.849	0.758	60.3%	80.0%	61.4%	79.3%	301.7%	49.6%
PVR + MLR	0.776 (0.705–0.836)	0.723	0.761	0.867	58.6%	90.9%	77.3%	80.6%	644.9%	45.5%
NLR + MLR	0.750 (0.677–0.813)	0.806	0.878	0.446	69.0%	78.2%	62.5%	82.7%	316.1%	39.7%

^*^Comparison with the AUC of the ESR; ^&^Comparison with the AUC of CRP; ^#^Comparison with the AUC of the ESR in combination with CRP; Multiple comparisons were conducted using the Bonferroni method to adjust the level of α (α/3 = 0.0167)

**Table 5:** Diagnostic value of combining all peripheral blood biomarkers in PJI

Different combination	AUC (95% CI)	*P* value*	*P* Value^&^	*P* value^#^	Sensitivity	Specificity	PPV	NPV	+ LR	-LR
ESR	0.760 (0.688–0.823)	-	0.971	0.152	63.8%	76.4%	58.7%	80.0%	270.3%	47.4%
CRP	0.758 (0.687–0.821)	0.971	-	0.483	79.3%	69.1%	57.5%	86.4%	256.6%	30.0%
ESR + CRP + PLR + PVR + NLR	0.834 (0.768–0.886)	**0.026**	0.058	0.086	62.1%	92.7%	81.8%	82.3%	853.8%	40.9%
ESR + CRP + PLR + PVR + MLR	0.824 (0.757–0.878)	**0.043**	0.115	0.154	84.5%	71.8%	61.3%	89.8%	299.8%	21.6%
ESR + CRP + PVR + NLR + MLR	0.826 (0.760–0.880)	**0.039**	0.101	0.134	87.9%	67.3%	58.6%	91.4%	268.7%	17.9%
CRP + PLR + PVR + NLR + MLR	0.837 (0.772–0.890)	**0.034**	**0.040**	0.086	87.9%	66.4%	58.0%	91.2%	261.4%	18.2%
ESR + CRP + PLR + PVR + NLR + MLR	0.841 (0.777–0.893)	**0.010**	**0.041**	**0.040**	81.0%	72.7%	61.0%	87.9%	297.1%	26.1%
WBC + ESR + CRP + PLR + PVR + NLR + MLR	0.851 (0.777–0.902)	**0.007**	**0.023**	**0.018**	77.6%	80.0%	67.2%	87.1%	388.0%	28.0%
PLT + ESR + CRP + PLR + PVR + NLR + MLR	0.839 (0.792–0.911)	**0.013**	**0.044**	**0.045**	89.7%	65.5%	57.8%	92.3%	259.6%	15.7%
All biomarkers combination										
WBC + PLT + ESR + CRP + PLR + PVR + NLR + MLR	0.853 (0.790–0.909)	**0.005**	**0.021**	**0.015**	82.8%	72.7%	61.5%	88.9%	303.5%	23.7%

^*^Comparison with the AUC of the ESR; ^&^Comparison with the AUC of CRP; ^#^Comparison with the AUC of the ESR in combination with CRP; Multiple comparisons were conducted using the Bonferroni method to adjust the level of α (α/3 = 0.0167)

### Comparison of peripheral blood biomarkers in the different PJI subgroups

Among the microbial cultures of 58 patients diagnosed with PJI, the most frequently detected pathogenic bacterium was *Staphylococcus epidermidis* (11/39, 28.21%), followed by *Staphylococcus aureus* (9/39, 23.08%). Upon conducting a subgroup analysis on these 58 PJI patients, it was observed that pathogenic microorganism culture had positive results in 39 patients and negative results in 19 patients. Additionally, within the cohort, 20 patients were diagnosed with acute PJI, while the remaining 38 presented with chronic PJI. A significant statistical difference was observed in the peripheral blood biomarkers, specifically the ESR and CRP levels, between the culture-negative and culture-positive groups (47.15 ± 28.50 vs 26.42 ± 8.01, *P* = 0.003; 33.22 ± 41.12 vs 9.02 ± 7.25, *P* = 0.014). Further analysis revealed that the only statistically significant difference in CRP levels existed between the acute PJI subgroup and chronic PJI subgroup (42.47 ± 55.12 vs 16.25 ± 12.65, *P* = 0.028) (Table 6).

**Table 6:** Comparison of peripheral blood biomarkers in the different PJI subgroups

Laboratory examination	Culture positive PJI (*N* = 39)	Culture negative PJI (*N* = 19)	*P* value	Acute PJI (*N* = 20)	Chronic PJI (*N* = 38)	*P* value
ESR (mm/h) (mean ± SD)	47.15 ± 28.50	26.42 ± 8.01	**0.003**	48.25 ± 37.15	36.21 ± 15.92	0.595
CRP (mg/L) (mean ± SD)	33.22 ± 41.12	9.02 ± 7.25	**0.014**	42.47 ± 55.12	16.25 ± 12.65	**0.028**
PLR (mean ± SD)	293.17 ± 198.31	208.85 ± 104.45	0.088	307.70 ± 209.37	243.36 ± 155.48	0.295
PVR (mean ± SD)	29.96 ± 9.88	28.26 ± 10.21	0.544	30.05 ± 11.61	29.07 ± 9.08	0.725
NLR (mean ± SD)	5.48 ± 3.01	4.08 ± 2.25	0.078	5.08 ± 2.93	4.98 ± 2.84	0.825
MLR (mean ± SD)	0.55 ± 0.30	0.50 ± 0.50	0.63	0.60 ± 0.36	0.50 ± 0.38	0.262

### ROC curves analysis for individual and combined peripheral blood biomarkers in acute PJI and chronic PJI group

**Fig. 6:**
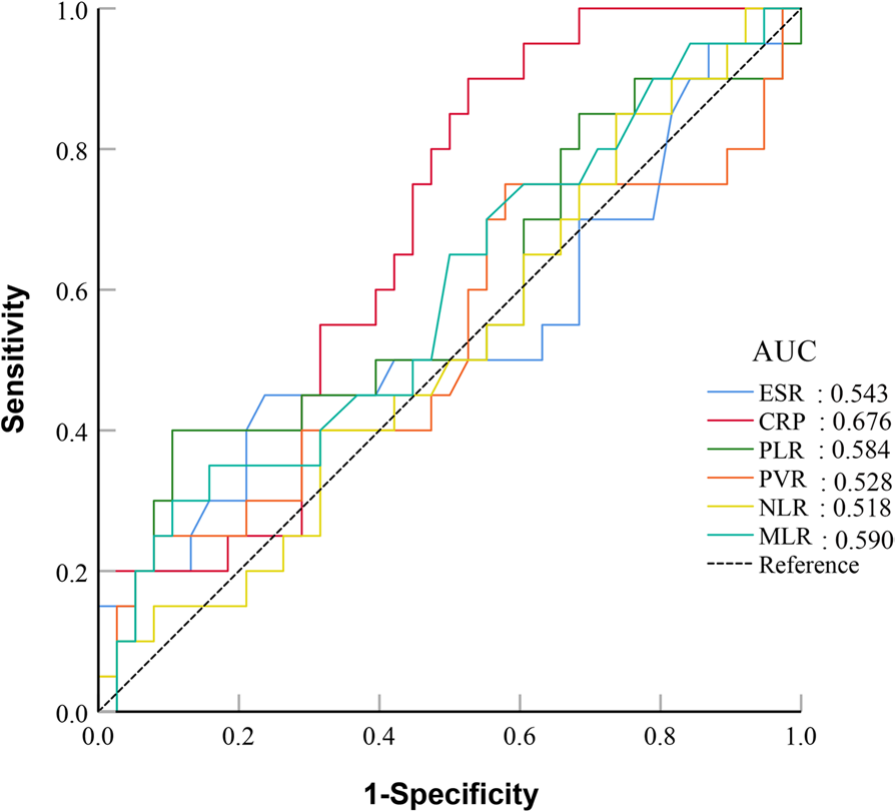
Results of ROC analysis of individual peripheral blood biomarkers in acute PJI and chronic PJI group

**Fig. 7:**
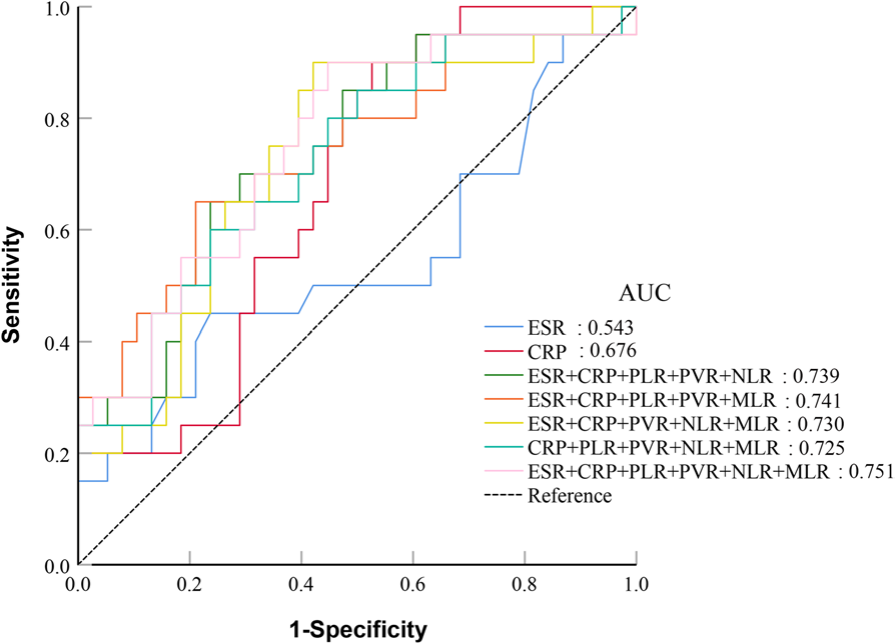
Results of the ROC analysis of patients in combination with five or all peripheral blood biomarkers in acute PJI and chronic PJI group

The ROC analysis of 20 patients with acute PJI and 38 patients with chronic PJI is shown in the Figs. 6 and 7.

The AUC ranked from highest to lowest were CRP (AUC = 0.676) > MLR (AUC = 0.590) > PLR (AUC = 0.584) > ESR (AUC = 0.543) > PVR (AUC = 0.528) NLR (AUC = 0.518). Our study results indicate that these different biomarkers have poor diagnostic values in diagnosing acute and chronic PJI (Fig. 6).

In comparing a range of combinations involving the ESR, CRP, PLR, PVR, NLR, and MLR in peripheral blood with ESR and CRP separately, the ROC curve results indicate that thoroughly combining all the biomarkers exhibits the highest AUC (0.751, 95%CI:0.620–0.855) in the subgroup. The novel combination demonstrates a favourable sensitivity of 90.0%. However, its specificity is only 55.3%. In contrast with individual and combined assessments of CRP and ESR, this new combined approach did not show substantial diagnostic value in the subgroup; the AUC results show that the diagnostic value is medium (Figs. 6 and 7, Table 7 and 8).

**Table 7:** Diagnostic value of individual peripheral blood biomarkers in acute PJI and chronic PJI group

Laboratory examination	AUC (95% CI)	Youden Index	Cut off	P* value	Sensitivity	Specificity	PPV	NPV	+ LR	-LR
ESR	0.543 (0.407–0.674)	0.213	42	-	45.0%	76.3%	189.9%	72.1%	50.0%	72.5%
CRP	0.676 (0.541–0.793)	0.374	10.8	0.122	90.0%	47.4%	171.1%	21.1%	47.4%	90.0%
PLR	0.584 (0.447–0.712)	0.295	350.45	0.594	40.0%	89.5%	381.0%	67.0%	66.7%	73.9%
PVR	0.528 (0.393–0.661)	0.171	23.8	0.887	75.0%	42.1%	129.5%	59.4%	40.5%	76.2%
NLR	0.518 (0.383–0.651)	0.111	2.52	0.779	85.0%	26.3%	115.3%	57.0%	37.8%	76.9%
MLR	0.590 (0.453–0.718)	0.195	0.73	0.478	30.0%	89.5%	285.7%	78.2%	60.1%	70.8%

*PPV* positive predictive value, *NPV* negative predictive value, + *LR* positive likelihood ratio, *-LR* negative likelihood ratio

^*^Comparison with the AUC of ESR

**Table 8:** Diagnostic value of combining five and all peripheral blood biomarkers in acute PJI and chronic PJI group

Different combination	AUC (95% CI)	*P* value*	*P* Value^&^	*P* value^#^	Sensitivity	Specificity	PPV	NPV	+ LR	-LR
ESR	0.543 (0.407–0.674)	-	0.122	0.196	45.0%	76.3%	189.9%	72.1%	50.0%	72.5%
CRP	0.676 (0.541–0.793)	0.122	-	0.666	90.0%	47.4%	171.1%	21.1%	47.4%	90.0%
ESR + CRP	0.696 (0.561–0.810)	0.196	0.666	-	70.0%	68.4%	221.5%	43.9%	53.8%	81.2%
**The combination of five biomarkers**										
ESR + CRP + PLR + PVR + NLR	0.739 (0.608–0.846)	0.063	0.299	0.410	65.0%	76.3%	274.3%	45.9%	59.1%	80.6%
ESR + CRP + PLR + PVR + MLR	0.741 (0.609–0.847)	0.067	0.304	0.415	65.0%	79.0%	309.5%	44.3%	62.0%	81.1%
ESR + CRP + PVR + NLR + MLR	0.730 (0.598–0.838)	0.077	0.325	0.487	90.0%	57.9%	213.8%	17.3%	52.9%	91.7%
CRP + PLR + PVR + NLR + MLR	0.725 (0.592–0.834)	**0.013**	0.439	0.728	60.0%	76.3%	253.2%	52.4%	57.1%	78.4%
**All biomarkers combination**										
ESR + CRP + PLR + PVR + NLR + MLR	0.751(0.620–0.855)	**0.039**	0.226	0.358	90.0%	55.3%	201.3%	18.1%	51.4%	91.3%

*PPV* positive predictive value, *NPV* negative predictive value, + *LR* positive likelihood ratio, *-LR* negative likelihood ratio, Multiple comparisons were conducted using the Bonferroni method to adjust the level of α (α/3 = 0.0167)

^*^Comparison with the AUC of ESR

## Discussion

In this retrospective study, we compared the different biomarker ratios in peripheral blood with traditional inflammatory biomarkers ESR and CRP to explore the potential value of these biomarkers in the diagnosis of PJI. The results confirmed significant differences in WBC, PLT, PLR, PVR, NLR, and MLR between PJI and AL patients. Still, the AUC value of the ROC curve showed that the diagnostic value of these different biomarker ratios in PJI is lower than ESR or CRP. Interestingly, when all ratios were combined with ESR and CRP, the diagnostic capability of this novel combination was significantly enhanced, surpassing the performance of ESR and CRP individually or in combination.

PJI is a severe postoperative syndrome that occurs after TJA, which has been identified as a crucial contributor to knee pain, dysfunction, disability, and even mortality following TKA [6, 19]. Studies have revealed that more than 25% of TKA revisions are attributable to PJI [20], underscoring the importance of timely diagnosis for effective treatment planning and optimal therapeutic outcomes. However, preoperative diagnosis of PJI is still difficult due to the emergence of infections caused by specific pathogens and bacterial biofilm formation [1–4]. Although diagnostic techniques such as leukocyte esterase (LE), ɑ-defensin, PCR, NGS and others have shown the potential to improve diagnostic accuracy, their high technical complexity and medical costs limit their widespread use [10–12, 21]. Against this backdrop, our study focused on the clinical utility of readily available and cost-effective blood routine biomarkers, specifically the PLR, PVR, NLR, and MLR, either individually or in combination with the classical serum markers ESR and CRP recommended in the MSIS guidelines, for aiding in the diagnosis of PJI. By comparing the AUC, sensitivity, and specificity of these parameters with those of the ESR and CRP, we aimed to provide initial insights into the potential value of these indicators in assisting PJI diagnosis.

The ESR and CRP are important biomarkers reflecting the presence of inflammation in the body. Due to their high sensitivity and ease of detection, they have become important screening tools for PJI [9]. In a study involving 156 patients, Yang et al. reported that the AUCs of ESR and CRP for assisting in diagnosing PJI were 0.822 and 0.901, with sensitivities of 70.2% and 91.2%, respectively, and specificities of 85.9% and 82.7%, respectively, confirming that both the ESR and CRP have good diagnostic performance for PJI [22]. However, our study results indicate that the clinical value of the ESR and CRP in the diagnosis of PJI is medium, with AUCs of 0.760 (95% CI: 0.688–0.823) and 0.758 (95% CI: 0.687–0.821), respectively. Wu et al. also reported the limited utility of the traditional biomarker CRP (AUC = 0.737) in diagnosing PJI [23]. Although the numerical changes in traditional biomarkers ESR and CRP are critical reference criteria for diagnosing PJI, their diagnostic performance is not entirely satisfactory due to limitations of many factors, such as sample size, disease progression, and different types of pathogens.

The PLR and PVR represent the ratio of platelet count to lymphocyte count and mean platelet volume, respectively, which can be used to reflect the degree of systemic inflammatory response and infection status [24, 25]. It has been reported that elevations in the PLR and PVR are closely associated with bacterial infections, possibly due to platelets being activated and promoting immune cell aggregation and the production of proinflammatory cytokines during the inflammatory response [25, 26]. These biomarkers offer a range of advantages, including simplicity, speed, affordability, and so on. A study by Wang et al. revealed that PVR is a valuable biomarker for the early diagnosis of infectious bone nonunion [27]. Recent investigations have also shown the potential of the PLR and PVR in aiding PJI diagnosis [28, 29]. In a retrospective analysis of 464 patients who underwent revision surgery (191 of whom had PJI), Klemt et al. demonstrated that the PLR and PVR were valuable diagnostic tools, with an AUC of 0.86 for both indicators. A cut-off value of 237.9 for the PLR yielded sensitivity and specificity rates of 75.9% and 82.8%, respectively, while a cut-off of 27.8 for PVR led to sensitivity and specificity rates of 86.4% and 75.5%, respectively [30]. However, Wang H et al. posited that the PLR and PVR are of limited use in diagnosing PJI, with an AUC of 0.700, sensitivity rates of 51.22% and 48.78%, and specificity rates of 80.58% and 86.33% for PLR and PVR, respectively [31]. Our study produced similar results to those of Wang H et al., indicating that PLR and PVR are not superior to the ESR in aiding PJI diagnosis, as their AUC and sensitivity values are lower compared to ESR and CRP. Although the specificity of PVR was greater than that of the ESR in our study (81.8% vs 76.4%), its sensitivity was only 51.7%, which greatly increases the risk of missed diagnosis.

The NLR and MLR offer insight into the absolute value alterations ofin neutrophils, lymphocytes, and monocytes; these changes can reflect respiratory system and urinary system infections and play important roles in the diagnosis of early infectious diseases, evaluation of infection severity and prediction of clinical prognosis [32, 33]. Our research team conducted a retrospective analysis of 168 patients and revealed significantly greater NLR and MLR values among PJI patients than those in the AL patients, with statistical significance (*P* < 0.001), suggesting that NLR and MLR can be used to assist in the diagnosis of PJI, which was consistent with the findings of Maimaiti et al. [24]. However, Xu et al. contend that the NLR and MLR have limited diagnostic potential for PJI due to their lower sensitivity and specificity compared to CRP, which implies that the NLR and MLR cannot replace the CRP as a diagnostic tool for PJI [34]. Similarly, Jiao et al. demonstrated that the NLR did not demonstrate a significant advantage in terms of sensitivity (73.58%) or specificity (70.97%) compared to the CRP or ESR [35]. Nonetheless, the results of Yu et al. and Zhao et al. indicate that the NLR is more valuable than the CRP and ESR in early PJI detection following TJA, which may be owing to the persistently high levels of CRP and the ESR in the early stages after joint replacement, while the NLR can return to preoperative levels relatively quickly [36, 37]. In our study, the AUC, sensitivity, and specificity of the NLR were 0.723, 62.1%, and 73.6%, respectively, which were all lower than ESR. These findings suggest that the NLR is less effective than the ESR in aiding PJI diagnosis and cannot be a substitute biomarker. It is noteworthy that MLR showed the highest specificity of 83.6% compared to the ESR and CRP in the diagnosis of PJI, but this was achieved by sacrificing the sensitivity of the MLR in diagnosing PJI.

Considering the limitations of the PLR, PVR, NLR and MLR in the diagnosis of PJI, our research team sought to enhance the diagnostic sensitivity and efficiency of PJI by integrating the measurements of WBC, PLT, ESR, CRP, PLR, PVR, NLR, and MLR. We observed that the novel combination of the WBC, PLT, ESR, CRP, PLR, PVR, NLR, and MLR had superior diagnostic performance with the highest AUC (0.853). Notably, statistical analysis revealed a significant difference between the novel combination and the ESR or ESR combined with CRP (*P* < 0.0167). Therefore, these findings indicate that by combining readily accessible and affordable peripheral blood biomarkers in routine blood test results, the accuracy of diagnosing PJI can be further enhanced.

### Limitations of this study

(I) This study is a single-center retrospective study and has a possible bias in patient information. (II) As a result of the small number of PJI patients included in this investigation, more extensive, prospective, and multicenter trials are necessary. (III) All patients were restricted to the Department of Joint Surgery in our hospital, and there were geographical and racial limitations.

## Conclusion

Our study suggests that changes in the level of inexpensive biomarkers such as the WBC, PLT, PLR, PVR, NLR and MLR in blood routine test are significantly different between PJI and AL groups. However, these biomarkers are not found to be superior to the classical markers ESR or CRP in diagnosing PJI and have limited value in this regard. On the other hand, when all these biomarkers are combined together with the ESR and CRP, a new combined model is formed. This combined model can effectively improve the diagnostic capability of PJI.

## Data Availability

The datasets generated and analyzed during the current study are available from the corresponding author on reasonable request.

## Abbreviations

PJI: Periprosthetic joint infection
TJA: Total joint arthroplasty
PLR: Platelet to lymphocyte ratio
PVR: Platelet count to mean platelet volume ratio
NLR: Neutrophil to lymphocyte ratio
MLR: Monocyte to lymphocyte ratio
TKA: Total knee arthroplasty
THA: Total hip arthroplasty
MSIS: The Musculoskeletal Infection Society
